# Supplementary figures and images for: Characterisation of the horse transcriptome from immunologically active tissues
Source: PeerJ. 2014 May 6;2:e382. doi: 10.7717/peerj.382 (PMC4017814; doi:10.7717/peerj.382)

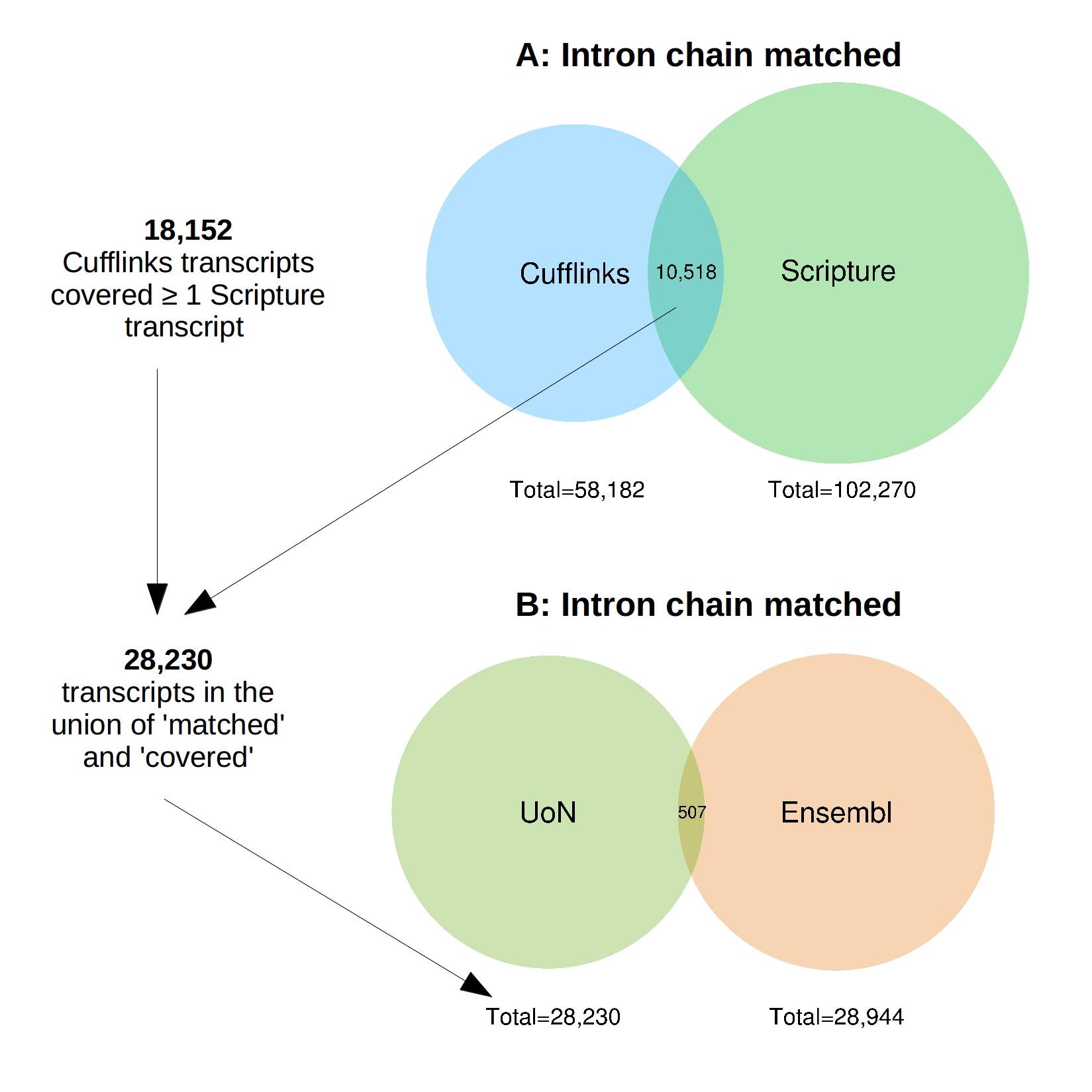

Supplement: File S9 — (A) Scripture assembled 102,270 stranded transcripts whereas Cufflinks reconstructed 58,182. The program “Cuffcompare” identified 10,518 transcripts where the intron chains completely matched. In addition to this 18,152 Cufflinks transcripts covered at least one Scripture transcript with the same compatible intron structure. The union of these two sets resulted in 28,230 transcripts. (B) The similarities between the 28,230 consensus “UoN” transcripts and the 28,944 Ensembl transcripts were compared and 507 transcripts were identified where the intron chains completely matched. The Venn diagrams were generated with R package “venneuler”. [file peerj-02-382-s009.jpg]
